# Low medication adherence and its associated factors among patients with type 2 diabetes mellitus attending Amana Hospital in Dar es Salaam, Tanzania: a cross-sectional study

**DOI:** 10.1093/inthealth/ihad042

**Published:** 2023-06-13

**Authors:** Irene F Doya, James J Yahaya, Advera I Ngaiza, Deogratius Bintabara

**Affiliations:** Department of Community Medicine, School of Medicine and Dentistry, University of Dodoma, Dodoma, Tanzania; Department of Pathology, School of Health Sciences, Soroti University, P. O. Box 211, Soroti, Uganda; Department of Pathology, Muhimbili University of Health and Allied Sciences, Dar es Salaam, Tanzania; Department of Pathology, Muhimbili National Hospital, Dar es Salaam, Tanzania; Department of Community Medicine, School of Medicine and Dentistry, University of Dodoma, Dodoma, Tanzania

**Keywords:** low medication adherence, risk factors, type 2 diabetes mellitus

## Abstract

**Background:**

Low medication adherence among patients with type 2 diabetes mellitus (T2DM) is associated with significant morbidity and mortality globally. We investigated the prevalence of low medication adherence and its associated factors among patients with T2DM.

**Methods:**

We used the Bengali version of the 8-item Morisky Medication Adherence Scale (MMAS-8) in measuring medication adherence among patients with T2DM who were attending the diabetes clinic at Amana Regional Referral Hospital in Dar es Salaam, Tanzania, from December 2021 to May 2022. Binary logistic regression analysis under multivariate analysis was used to determine the predictors of low medication adherence after controlling for confounders. A two-tailed p-value <0.05 was considered significant.

**Results:**

The prevalence of low medication adherence was 36.7% (91/248) of the subjects included in the study. Lack of formal education (adjusted odds ratio [AOR] 5.3 [95% confidence interval {CI} 1.717 to 16.312], p=0.004), having comorbidities (AOR 2.1 [95% CI 1.134 to 3.949], p=0.019) and drinking alcohol (AOR 3.5 [95% CI 1.603 to 7.650], p=0.031) were the independent predictors of low medication adherence.

**Conclusion:**

More than one-third of the patients with T2DM in this study had low medication adherence. Our study also showed that a lack of formal education, having comorbidities and drinking alcohol were significantly associated with low medication adherence.

## Background

Diabetes mellitus (DM) is a group of metabolic diseases characterized by long-standing hyperglycaemia resulting from defects in insulin production, insulin action and sometimes both.^1^ Type 2 diabetes mellitus (T2DM), which is a type of DM, is an important and growing health problem of public concern globally.^2^ The number of adults estimated to be living with T2DM in the sub-Saharan Africa (SSA) region in 2017 was 15.5 million, with a regional prevalence of about 6%.^3^ Studies have shown great variations in the prevalence of T2DM globally, mainly due to differences in lifestyle behaviours, including physical inactivity, diet, smoking, alcoholism and genetic factors.^4,5^ For example, in Ghana, the prevalence of T2DM in the general population was estimated to range between 3.3 and 6% and the prevalence was found to increase with age, with the vast majority of patients being from urban areas.^6^ In a study that was done in Algeria, the prevalence of T2DM was found to be 12.3%,^7^ while in Tanzania the prevalence of T2DM was found to be 7.8%, with the number of cases increasing with age.^8^

The management of patients with T2DM involves long-term use of anti-diabetic medications. Although studies have shown the advantages of using anti-diabetic medications in treating patients with T2DM, medication adherence in these patients is still a challenge. For example, the prevalence of low medication adherence among patients with T2DM in a study done in Tanzania was 34.3%.^9^ In another study in Botswana, the prevalence of low medication adherence of anti-diabetic medications was 41.8% among patients with T2DM.^10^ In 2021, Islam et al.^11^ reported a prevalence of low medication adherence of 48.2% among patients with T2DM in Bangladesh. Low medication adherence is indeed a devastating problem in patients with T2DM and studies have shown that the problem usually increases with the duration after treatment initiation.^12–14^

Some sociodemographic characteristics (young age and low income) and lifestyle behaviours (alcoholism, diet and smoking) may influence medication adherence, either positively or negatively.^13^ Other contributing factors to low medication adherence include poor medical facilities, lack of health insurance and comorbidities like hypertension, which usually increase health expenses.^9^ The most studied lifestyle behaviour is alcohol consumption, which affects T2DM in two ways: it directly affects glycaemic control by inducing hypoglycaemia^15^ and it influences suboptimal medication adherence.^16^

Poor or low medication adherence among patients with T2DM is associated with poor glycaemic control and decreased benefits from their prescribed medication. These can contribute to increased morbidity and mortality, development of comorbidities, poor quality of life and increased costs for healthcare.^13,17,18^

Little data are available regarding prevalence and its factors associated with low medication adherence among patients with T2DM in Tanzania. This creates a gap in knowledge and practices regarding care and management of this group of patients, causing poor glycaemic control as well as increased morbidity and mortality. This study aimed to assess the prevalence of low medication adherence and its associated factors among patients with T2DM in Tanzania.

## Methods

### Study design, setting and duration

This was a cross-sectional analytical hospital-based study. The study was conducted at Amana Regional Referral Hospital (ARRH) in the Ilala Municipal Council, Dar es Salaam, Tanzania, from December 2021 to May 2022. Ilala is one of the six municipal councils of the Dar es Salaam region, which is the main city and business centre in Tanzania and is located on the Indian ocean. ARRH has a capacity of 340 beds with a total number of outpatients attending per year ranging from 292 000 to 438 000. The clinic for patients with diabetes is conducted twice per week and approximately 20 new patients with T2DM are registered per clinic, for a total of about 2080 patients with T2DM recorded at the health facility per year.

### Patients’ characteristics and recruitment procedure

We included all patients with T2DM ≥18 y of age that were attending the diabetes clinic at the study site. All patients with T1DM and those who were seriously ill of debilitated were excluded from the analysis.

### Sample size estimation and sampling method

The sample size was calculated using the standard formula as described by Kish^19^ for prevalence of a single population: n=Z^2^p(100−p)/e^2^, assuming a 95% confidence interval (CI), standard normal variables (z score) of 1.96 with a margin of error (e) of 5% and a proportion (p) of 60.2% for medication adherence among patients with T2DM from a previous study.^20^ In order to overcome selection bias, we applied a lottery simple random sampling method to obtain the study participants from the study population of 503 patients with T2DM. Slips of paper with numbers from 1 through 503 were placed in a box and patients who had attended the diabetes clinic were requested to select one slip of paper from the box. All participants who picked odd numbers were included in the analysis. The process was done consecutively until the required sample size of 248 was obtained.

### Assessment of medication adherence

We adapted the Bengali version of the 8-item Morisky Medication Adherence Scale (MMAS-8) questionnaire^11^ to measure medication adherence in our study. Total scores on the MMAS-8 range from 0 to 8. The questions are answered with ‘yes’ or ‘no’. Because the eight questions are negatively coded items, 0 is given to a ‘yes’ response and 1 is given to a ‘no’ response. The scores are categorized as follows: 8 points, high medication adherence; 6–7 points, moderate medication adherence; and 0–5 points, low medication adherence. Internal consistency was examined and Cronbach's α coefficient was 0.83, which was obtained after conducting a pilot study that included 20 patients with T2DM from a different health facility.

### Physical activity

In this study, physical activity was categorized as was done in the previous study.^21^ Patients who reported having physical activity for <30 min, 30–45 min and >45 min per week were considered to have mild, moderate and vigorous physical activity, respectively.

### Diet

Diet for patients with T2DM was defined as small and frequent (≥5 meals/day), containing fruits, vegetables, high fibre, whole grains and low in sugar, as was defined previously.^22^ A total of 10 items were used to measure adherence to the recommended diet using a Likert scale with the following scores: eating always, 2; eating sometimes, 1; eating never, 0). The total score ranged from 0 to 20 points and was categorized as follows: 15–20, good adherence to dietary recommendations; 10–15, moderate adherence to dietary recommendations; and <10, non-adherent to dietary recommendations.^23^

### Data collection

We used a semi-structured questionnaire and face-to-face interviews to collect data from study participants. The questionnaire included questions that were adapted from a previous study of the MMAS-8 in measuring medication adherence.^11^ Data were collected in a separate room within the diabetes clinic to maintain the privacy of patients. The questionnaire was first pre-tested among 20 T2DM patients from a different hospital. Each patient was interviewed for approximately 20 min followed by signing a written informed consent.

### Statistical analysis

Data were analysed using SPSS version 25.0 (IBM, Armonk, NY, USA). Because the number of low and moderate scores was low, we combined them to make the low medication adherence group (MMAS 0–5 and 6–7 points), with the other group for the patients with high medication adherence (MMAS 8 points). We used binary logistic regression analysis to determine the predictors of low medication adherence under multivariate analysis after controlling for independent variables (age of the patient, level of education, residence, occupation, health insurance, family income, comorbidities, duration of T2DM, route of medication and drinking alcohol ). A two-tailed p-value <0.05 was considered significant.

## Results

### Sociodemographic characteristics

The sociodemographic characteristics of the patients are shown in Table 1. The study included a total of 248 patients with T2DM with a mean age of 59.8±12.1 y. The majority (70.2% [174/248]) of the participants were females and 38.3% (95/248) of the patients were obese, with a mean body mass index (BMI) of 28.3±6 kg/m^2^. A total of 16.1% (40/248) of participants had a family income below the International Poverty Line (<US$1.9/day). Also, 35.5% (88/248) of the patients had attained a tertiary level of education (Table 1).

**Table 1.** Sociodemographic characteristics of the patients with T2DM (N=248)

Variables	Frequency (n)	Percent (%)
Age (years)		
<45	20	8.1
≥45	228	91.9
BMI (kg/m^2^)		
Normal (18.5–24.9)	66	26.6
Underweight (<18.5)	7	2.8
Overweight (25–29.9)	80	32.3
Obese (≥30)	95	38.3
Sex		
Male	74	29.8
Female	174	70.2
Marital status		
Single	4	1.6
Married/cohabiting	152	61.3
Divorced/separated	15	6.0
Widower/widowed	77	31.1
Education level		
Informal	52	21.0
Primary	38	15.3
Secondary	70	28.2
Tertiary	88	35.5
Residence		
Urban	240	96.8
Rural	8	3.2
Religion		
Muslim	79	31.9
Christian	159	64.1
None	10	4.0
Occupation		
Employed	72	29.0
Self-employed	79	31.9
Unemployed	97	39.1
Level of income (TZS) per month		
<132 810	40	16.1
132 810–500 000	59	23.8
>500 000	149	60.1

### Clinical characteristics and lifestyle behaviours of the study patients

The vast majority (91.1% [226/248]) of the patients were using an oral route for taking their medications and the majority (82.3% [204/248]) of patients were on oral metformin. Regarding lifestyle behaviours of the patients, 14.5% (36/248) and 2.8% (7/248) were drinking alcohol and smoking, respectively. Also, 66.9% (166/248) of the T2DM patients had good dietary intake adherence (Table 2).

**Table 2.** Clinical characteristics and lifestyle behaviours of the study patients (N=248)

	Frequency	Percentage
Variables	(n)	(%)
Family history of DM		
Yes	48	19.4
No	200	80.6
Health insurance		
Insured	179	72.2
Not insured	69	27.8
Comorbidities		
Yes	154	62.1
No	94	37.9
Duration of T2DM (years)		
≤5	78	31.5
>5	170	68.5
Route of medication		
Oral	191	77.0
Injectables	33	13.3
Mixed	24	9.7
Current medications		
Metformin	204	82.3
Insulin	43	17.3
Gemer 1	1	0.4
Alcoholism		
Yes	36	14.5
No	212	85.5
Smoking		
Yes	7	2.8
No	241	97.2
Physical activity (minutes per week)		
<30 (mild)	21	8.5
30–45 (moderate)	22	8.9
>45 (vigorous)	7	2.8
None	198	79.8
Dietary intake adherence		
Good adherence	100	40.3
Moderate adherence	66	26.6
Non-adherent	82	33.1

### Medication adherence among T2DM patients

Table 3 presents the frequency of the ‘no’ responses for the MMAS-8. The majority of the study participants (63.3% [157/248]) had high medication adherence (MMAS score 8) followed 26.2% (65/248) of participants with moderate medication adherence (MMAS score 6–7) and 10.5% (26/248) had low medication adherence (MMAS score 0–5). Thus the percentage of patients with low medication adherence in this study was 36.7% (91/248).

**Table 3.** The MMAS-8

Item	Patients answering ‘no’, n (%)
1. Do you sometimes forget to take your anti-diabetes medicines?	
2. Over the past 2 weeks, were there any days when you did not take your anti-diabetes medicines?	210 (84.7)
3. Have you ever stopped taking your anti-diabetes medicines without telling your physician because you felt worse after taking them?	219 (88.4)
4. When you travel or leave home, do you sometimes forget to pack your anti-diabetes medicines?	163 (65.7)
5. Did you take your anti-diabetes medicines yesterday?	205 (82.7)
6. When you feel like your blood sugar is under control, do you sometimes stop taking your anti-diabetes medicines?	220 (88.7)
7. Taking medications daily is a real inconvenience for some patients. Do you ever feel hassled about sticking to your T2DM treatment plan?	231 (93.1)
8. How often do you have difficulty remembering to take your anti-diabetes medicines?	216 (87.1)
Distribution of scores:	
0–5 (low medication adherence)	26 (10.5)
6–7 (moderate medication adherence)	65 (26.2)
8 (high medication adherence)	157 (63.3)

### Binary logistic regression analysis for predictors of low medication adherence

In the univariate or bivariate analysis, both patients with primary school (unadjusted odds ratio [UOR] 0.5 [95% CI 1.104 to 5.825], p=0.028) and secondary school (UOR 0.9 [95% CI 1.446 to 5.883], p=0.003) were significantly protected against having low medication adherence. Moreover, those who had informal education were 4.5 times more likely to have low medication adherence compared with those with a tertiary education (95% CI 2.139 to 9.624, p<0.001). Unemployed patients were twice as likely to have low medication adherence compared with those who were employed (95% CI 1.036 to 3.967, p=0.039). Also, patients who had no health insurance were 2.3 times more likely to have low medication adherence compared with patients who had health insurance (95% CI 1.281 to 3.990, p=0.005).

Patients who had any comorbidity were 1.4 times more likely to have low medication adherence than those who had no comorbidities (95% CI 0.235 to 0.741, p=0.003). Also, patients with T2DM who had ≤5 y since diagnosis half as likely to have low medication adherence compared with patients with >5 years since diagnosis (UOR 0.5 [95% CI 0.551 to 2.510], p=0.04). Alcoholic patients with T2DM were 3.3 times more likely to have low medication adherence (95% CI 1.571 to 6.752, p=0.002). Self-employment, age, level of income and route of taking medication were not associated with low medication adherence.

Under multivariate analysis, having a primary level of education was significantly negatively associated with low medication adherence (adjusted odds ratio [AOR] 0.8 [95% CI 1.129 to 6.780], p=0.026). Patients with T2DM who had informal education were 5.3 times more likely to have low medication adherence than patients who had a tertiary level of education (95% CI 1.717, p=0.004). Patients with T2DM who had comorbidities were also 2.1 times more likely to have low medication adherence than patients who had no comorbidities (95% CI 1.134 to 3.949, p=0.019). Also, alcoholic patients were 3.5 times more likely to have low medication adherence compared with non-alcohol-drinking patients (95% CI 1.603 to 7.650, p=0.031) (Table 4).

**Table 4.** Determination of predictors of low medication adherence among patients with T2DM

Variables	Univariate analysis, UOR (95% CI), p-value	Multivariate analysis, AOR (95% CI), p-value
Age (years)		
<45	0.6 (0.330 to 1.379), 0.243	–
≥45	[Reference]	
Level of education		
Informal	4.5 (2.139 to 9.624), 0.000	5.3 (1.717 to 16.312), 0.004*
Primary	0.5 (1.104 to 5.825), 0.028	0.7 (0.859 to 8.282), 0.090
Secondary	0.9 (1.446 to 5.883), 0.003	0.8 (1.129 to 6.780), 0.026*
Tertiary	[Reference]	[Reference]
Place of residence		
Urban	[Reference]	[Reference]
Rural	3.6 (0.884 to 14.856), 0.074	1.4 (0.085 to 1.835), 0.236
Occupation		
Employed	[Reference]	[Reference]
Self-employed	2.0 (0.991 to 3.982), 0.053	1.9 (0.637 to 5.376), 0.258
Unemployed	2.0 (1.036 to 3.967), 0.039	1.5 (0.665 to 3.242), 0.342
Health insurance		
Insured	[Reference]	[Reference]
Not insured	2.3 (1.281 to 3.990), 0.005	1.7 (0.338 to 1.296), 0.229
Family income (TZS/month)		
<132 810	1.4 (0.689 to 2.987), 0.335	–
132 810–500 000	0.7 (0.309 to 1.230), 0.210	–
>500 000	[Reference]	
Comorbidities		
Yes	1.4 (0.235 to 0.741), 0.003	2.1 (1.134 to 3.949), 0.019*
No	[Reference]	[Reference]
Duration of T2DM (years)		
≤5	0.5 (0.551 to 2.510), 0.040	0.6 (0.299 to 1.241), 0.172
>5	[Reference]	[Reference]
Route of medication		
Oral	[Reference]	–
Injectables	1.2 (0.551 to 2.510), 0.676	–
Mixed	1.3 (0.545 to 3.065), 0.561	–
Taking alcohol		
Yes	3.3 (1.571 to 6.752), 0.002	3.5 (1.603 to 7.650), 0.031*
No	[Reference]	

Adjusted independent factors include age of the patient, level of education, residence, occupation, health insurance, family income, comorbidities, duration of T2DM, route of medication and drinking alcohol.

*Statistically significant findings.

## Discussion

Medication adherence among T2DM patients plays a critical role in glycaemic control, which in turn can contribute to increased morbidity and mortality. Over a period of 8 y since the last two studies were done in Tanzania addressing this subject,^20,24^ there is still a significant percentage of T2DM patients with low medication adherence. This study found that more than one-third of T2DM patients have low medication adherence. A lack of formal education, drinking alcohol and having comorbidities were the independent predictors of low medication adherence in this study.

The prevalence of low medication adherence of 36.7% in this study was close to the 36.9% and 39.8% reported in China^25^ and Tanzania,^20^ respectively, but higher than the 27% that was reported in the USA.^26^ Higher prevalences of low medication adherence of 42.8%, 45.2% and 61.1% have been reported in Bangladesh,^11^ Ethiopia^27^ and Saudi Arabia,^28^, respectively. The difference in methodology used for the compared studies may explain the discrepancies in the prevalence of low medication adherence. For instance, the use of four items (questions) in measuring medication adherence commonly seen when using the MMAS-4 has less ability to discriminate between medication adherence and non-adherence compared with the MMAS-8.^29^

Also, differences in the sociodemographic characteristics of the study subjects, including disease-related issues such as duration of T2DM since diagnosis, could also explain differences in the prevalence of low medication adherence. For example, Rwegerera^20^ reported that elderly T2DM patients are more likely to have poor medication adherence to anti-diabetic drugs than young patients. However, this seems to contrast with findings from studies in which it was found that younger patients with T2DM were positively associated with poor medication adherence.^13,30^

Both modifiable and non-modifiable factors affect medication adherence among patients with chronic diseases such as T2DM either positively or negatively. For example, it has been proven that excessive alcohol intake not only negatively impacts adherence of anti-diabetic medications, but also accelerates progression of the disease, including the inability to control blood sugar.^31^ Patients with T2DM who are alcoholic are more likely to have low medication adherence than non-alcoholic patients, which may contribute to increased morbidity and mortality.^28,30,31^ This is in line with our findings in which alcoholic patients were almost 4 times more likely to have low medication adherence compared with non-alcoholic patients. Indeed, alcoholism is associated with detrimental health behaviours, and previous studies have shown that alcohol use has an inverse relationship with the frequency of patient hospital visits, including poor medication adherence.^13,33^

Comorbidities such as hypertension, cardiovascular disease and chronic kidney disease usually develop in patients with T2DM over a period of time. These diseases tend to compromise the ability of patients to attend regular hospital visits and even fail to adhere to the treatment regimens prescribed for them. In previous studies, it was shown that the presence of comorbidities among patients with T2DM was significantly associated with low medication adherence.^34–36^ This is in agreement with our findings, as T2DM patients who had comorbidities were 2 times more likely to have low medication adherence compared with patients had no comorbidities. Also, Ayele et al.^36^ reported that having comorbidities among patients with T2DM was associated with a 32% reduction in having good medication adherence. The presence of additional chronic comorbidity has an impact on the treatment and management of T2DM.^37^ The Medical Expenditure Panel Survey reported that most adults with DM have at least one comorbidity, often resulting in multiple prescriptions with a variety of complex drug regimens and polypharmacy, which affects compliance in diabetic patients.^38^

For patients with T2DM, it has been shown that their academic achievement has a positive association with medication adherence. In three previous studies it was reported that informal education was associated with low medication adherence.^39–41^ This is also in keeping with the findings in our study in which we observed that patients who had informal education were 5 times more likely to have poor medication adherence. Having a low level of education is more likely to be associated with other issues that may impact medication adherence, including low income, local beliefs and social and psychological perspectives that together may act as a barrier towards completion of hospital visits and medical adherence.

Affordability of anti-diabetic drugs among patients with T2DM may sometimes be challenging due to financial constraints and poverty. This may contribute to irregular or low medication adherence among patients with T2DM. Subsidized health insurance among poor patients with T2DM has been shown to be associated with increased medication adherence. For example, Datta and Fazlul,^42^ in the USA, reported that the subsidized insurance under the Affordable Care Act helped to increase medication adherence significantly. Two studies in Nigeria^14^ and the United Arab Emirates^12^ reported a positive association between medication adherence and having health insurance among patients with T2DM. Also, in our study we found that patients who had no health insurance had a high chance of having low medication adherence compared with patients who had health insurance, although the difference did not reach statistical significance.

## Study limitations

This study had the following limitations. We were unable to identify some of the patients that might have diabetes knowledge prior interviewing them. This might have influenced their ability to answer the questions included in the questionnaire. By relying on self-reported responses by the patients, there may have been some information bias, as the patients might have given responses to make them appear responsible for their own health and with good health seeking behaviours, which may not necessarily be true.

## Conclusions

Although the majority of patients showed good medication adherence, more than one-third of the patients did have low medication adherence. This low medication adherence was significantly associated with a lack of formal education, drinking alcohol and having comorbidities. The patients with low medication adherence are at a high risk of morbidity and mortality, necessitating stringent measures including providing diabetes education for these patients as well as the general population on modifiable factors such as alcoholism.

## Data Availability

The dataset used for this study is restricted by the Ethical Research Committee of the University of Dodoma, as if contains sensitive patient information. However, it can be accessed upon reasonable request from the Directorate of Research Publication and Consultancy, University of Dodoma, P.O. Box 259, Dodoma, Tanzania (drpc@udom.ac.tz).
